# Relationship of Physical Activity Levels and Body Composition with Psychomotor Performance and Strength in Men

**DOI:** 10.3390/healthcare13151789

**Published:** 2025-07-23

**Authors:** José Manuel Delfa-de-la-Morena, Pedro Pinheiro Paes, Frederico Camarotti Júnior, Rubem Cordeiro Feitosa, Débora Priscila Lima de Oliveira, Juan-José Mijarra-Murillo, Miriam García-González, Víctor Riquelme-Aguado

**Affiliations:** 1 Department of Physical Therapy, Occupational Therapy, Rehabilitation and Physical Medicine, Health Sciences Faculty, Rey Juan Carlos University, 28922 Madrid, Spain; jose.delfa@urjc.es (J.M.D.-d.-l.-M.); juanjose.mijarra@urjc.es (J.-J.M.-M.); 2 Department of Physical Education, Federal University of Pernambuco, Recife 50670-901, Brazil; pppaes@ufpe.br (P.P.P.); frederico.camarottijunior@ufpe.br (F.C.J.); rubem.cordeiro@ufpe.br (R.C.F.); debora.limaoliveira@ufpe.br (D.P.L.d.O.); 3 Department of Basic Health Sciences, Health Sciences Faculty, Rey Juan Carlos University, 28922 Madrid, Spain; victor.riquelme@urjc.es

**Keywords:** body fat, functional capacity, metabolic equivalent of task, motor skills, muscle strength

## Abstract

**Objective**: The objective of the study was to investigate the relationship between the level of physical activity and body composition, and the levels of motor skills and strength in overweight and obese men. **Methods**: The research involved 64 men. Body composition, physical activity, motor control, Motor Control Test (MCT), and strength variables were evaluated. Body composition was assessed by DXA, and the participants were classified into two groups according to the percentage of total fat mass: greater and less than 27.65%. Physical activity was assessed using accelerometry, and motor control was measured with posturography, which provided a composite score of motor performance and postural control effectiveness. Strength was assessed using hand, leg, and back dynamometers. **Results**: The participants with a higher percentage of body fat had a lower DSI (Dynamic Strength Index) (*p* < 0.001) and significantly reduced PAL (physical activity level) and energy expenditure (*p* < 0.001). No significant differences were found in the muscle strength of the upper limbs (*p* = 0.06) and lower limbs (*p* = 0.419). With regard to MCT, there was a significant difference between groups in the backward direction (*p* = 0.041), with the group with the highest percentage of body fat showing lower values. **Conclusions**: Individuals with a higher percentage of body fat tend to have lower levels of strength, physical activity, and energy expenditure, which can lead to impaired balance. The findings highlight the need for targeted interventions to improve body composition and levels of strength and physical activity, with a positive impact on general health and quality of life. Emphasis should be placed on improving physical activity levels in male individuals with a higher percentage of fat mass to improve their body composition and dynamic strength levels, which are beneficial to life, particularly to help improve postural control.

## 1. Introduction

Technological advances in recent decades have contributed to a hypokinetic lifestyle, characterized by a low physical activity level (PAL). This behavior pattern, coupled with inadequate dietary habits, has intensified cases of malnutrition, overweight, and obesity worldwide [1]. These factors compromise physical capacities such as flexibility, strength, speed, endurance, and motor coordination, affecting motor skills across all age groups [2].

In the review conducted by Khodadad Kashi et al. [3], muscle strength (understood as the tension generated by a muscle in the face of opposing resistance) is recognized as an essential physical capacity for human health, contributing to good bone mineral density, healthy body composition (more muscle and less fat), and maintaining good body stability over the years.

With the reduction in muscle strength and impaired coordination, there is an increased risk of chronic diseases, loss of functionality, and a higher incidence of falls and injuries, particularly in individuals with overweight or obesity [4]. Muscles are metabolically active body tissue, meaning that the body uses energy to maintain them [3]. In this context, muscle strength and motor coordination are essential for health and functionality maintenance, having a direct influence on independence and quality of life [5]. PAL acts as a protective factor, promoting physical and mental well-being and reinforcing the importance of these components for a healthy life [6].

Nutritional status, generally measured by Body Mass Index (BMI) as well as body fat percentage, and PAL are important determinants for strength and motor coordination capacity [7]. While individuals with elevated BMI face mobility limitations and joint overload, physically active individuals tend to exhibit better motor performance [8]. Studies associate regular physical activity with higher levels of coordination and strength, showing that these factors influence physical functionality [9,10]. However, in recent years, the world’s adult population has been decreasing its PAL in parallel with the growth in the numbers of overweight and obese people [11].

Metabolic Equivalent of Task (MET) is introduced to indicate functional capacity and energy expenditure. Representing oxygen consumption at rest, the MET allows for the differentiation of activities of varying intensities and identifies the impact on the cardiovascular system and general well-being [12]. Higher MET levels have been linked to lower mortality rates and improved cardiovascular health, especially among groups with different PAL and nutritional profiles [13,14].

However, there is still a significant gap in the literature regarding an integrated understanding of the relationship between nutritional status, physical activity, strength, and motor coordination in adults. Many studies tend to look at these components in isolation, which limits the understanding of how their interactions can impact functional capacity, the risk of developing chronic non-communicable diseases, and, consequently, longevity with quality of life [15,16,17]. Analyzing these factors together would provide a broader and more appropriate perspective on health and its multidimensionality, offering greater support for the development of preventive and effective health promotion interventions in the public and clinical spheres.

Thus, the present study aimed to investigate the relationship between PAL and body composition on the levels of psychomotor performance and strength in men. The hypothesis is that people who are more physically active have better levels of strength and psychomotor performance and a lower percentage of fat. The outcomes of this study may aid in the creation of targeted intervention strategies, fostering the promotion of an active lifestyle and the improvement of functionality and quality of life in adults.

The hypothesis of this study is that physical activity levels and body composition have a significant relationship with psychomotor performance and strength in men. Individuals with higher body fat percentage are expected to have lower levels of strength, physical activity, and energy expenditure, which may negatively affect their balance and motor performance. This study differs from previous work by investigating the relationship between nutritional status, physical activity, strength, and motor coordination in adults in an integrated manner. While many studies tend to address these components in isolation, this study provides a broader perspective on physical health, offering further support for the development of preventive and health-promoting interventions.

## 2. Materials and Methods

### 2.1. Study Design

This observational, cross-sectional study employed a consecutive, non-probabilistic sampling strategy. All the participants received detailed written information about the objectives and procedures of the study and provided informed consent prior to participation. The study protocol was approved by the Ethics Committee of Rey Juan Carlos University and complied with the principles outlined in the Declaration of Helsinki for research involving human subjects.

### 2.2. Participants

The final sample consisted of 64 male participants aged 25 to 60 years, all of whom maintained stable body weight—defined as no weight fluctuation greater than ±2 kg in the preceding three months. The exclusion criteria included the presence of serious medical conditions, current or recent smoking (within the past six months), alcohol consumption, a history of balance disorders, prior knee or hip replacement surgery, lower limb trauma in the past six months, and diagnoses of arthritis or other severe inflammatory conditions affecting the lower limbs. Recruitment was conducted via email invitation to 325 individuals previously enrolled in the PRONAF study (Nutritional and Physical Activity Program for Obesity Control). Of these, 131 expressed interest and 67 met the eligibility criteria. Three participants later withdrew for personal reasons, yielding a final cohort of 64 individuals. The distribution between Groups 1 and 2 was based on the BMI values obtained. The median value for the participants was calculated as 27.65%.

### 2.3. Study Variables

#### 2.3.1. Body Composition

Body composition was assessed using dual-energy X-ray absorptiometry (DXA) (GE HealthCare Inc., Chicago, IL, USA) [18]. Among the available parameters, only total body fat percentage was recorded and analyzed, as it was considered the most relevant indicator for addressing the study objective related to adiposity and physical performance. Body fat percentage is a physiologically relevant indicator that has been widely associated with metabolic health, physical function, and motor performance, as supported by the current literature [3,4].

#### 2.3.2. Physical Activity

Physical activity was assessed using accelerometry with the SenseWear^®^ Armband (SWA) (Pittsburgh, PA, USA), a multisensory, objective device that is valid and reliable for measuring physical activity [19,20]. The PAL parameter [21] was used for this study. Among the parameters provided by the device, the physical activity level (PAL) was selected for this study. PAL is defined as the ratio of total daily energy expenditure (TDEE) to resting energy expenditure (REE) and is expressed as a dimensionless value. Since PAL reflects the average metabolic demand throughout the day, it is equivalent to the mean METs (Metabolic Equivalents of Task) over a 24 h period. Therefore, PAL values in this study were used as direct indicators of daily physical activity intensity, allowing for standardized comparisons between groups [21].

#### 2.3.3. Motor Control

The Motor Control Test (MCT) assesses the body’s automatic postural responses to rapid platform movements by measuring the neuromuscular response time across varying perturbation intensities and directions [22]. In this study, the MCT was conducted using the NeuroCom^®^ Smart Balance Master system (NeuroCom International, Clackamas, OR, USA), a computerized dynamic posturography platform that provides objective and reliable measurements of balance control. Prior to testing, the device was calibrated according to the manufacturer’s specifications to ensure precision in force plate sensitivity and platform displacement.

The participants stood barefoot on the platform with arms relaxed by their sides and were instructed to maintain their balance during a series of unexpected forward and backward platform translations. The protocol included three randomized trials at each perturbation level (small, medium, and large) to minimize anticipation effects. The MCT evaluates key parameters such as the latency of muscle response and displacement amplitude during these perturbations. The system generates a Composite MCT score, which integrates response times and movement amplitudes across all directions and intensities to produce a global indicator of postural control effectiveness.

In addition to the raw composite score, a standardized Z-score (Z MCT) was calculated based on normative values adjusted for age and height. This Z-score reflects how many standard deviations a participant’s performance deviates from the reference population, providing a normalized measure of postural control efficiency.

#### 2.3.4. Strenght

The Dynamic Strength Index (DSI) was obtained by measuring muscle strength using a Tecsymp Tkk5002 hand dynamometer and a Tecsymp Tkk5401 leg and back dynamometer (Tecsymp, Barcelona, Spain). The DSI value was calculated by adding up the results from both devices and dividing the total by the individual’s body weight.

### 2.4. Statistical Analysis

The statistical analysis was conducted using the Statistical Package for the Social Sciences (SPSS), version 20.0 (SPSS Inc., Chicago, IL, USA). The Shapiro—Wilk test was used to assess the normality of the data. Variables with normal distribution were analyzed using unpaired Student’s *t*-tests to compare individuals classified as sedentary or non-sedentary, and one-way ANOVA was applied to compare individuals categorized by BMI (normal weight, overweight, and obesity). For balance-related variables that did not follow a normal distribution, the Mann—Whitney U test was used. The results are reported as mean ± standard deviation. Additionally, effect sizes were calculated using Cohen’s d to quantify the magnitude of between-group differences, and 95% confidence intervals were reported to provide precision estimates for all comparisons. The significance level was set at *p* < 0.05.

## 3. Results

In this study, the median percentage of body fat among the participants was determined, establishing a cut-off point of 27.65%. Based on this value, the subjects were classified into two groups: Group 1, made up of individuals with a total body fat percentage of less than 27.65%, and Group 2, made up of those with a percentage above this limit. When analyzing sociodemographic characteristics, no significant differences were found between the groups (*p* = 0.449). However, body weight was significantly higher in Group 2 compared to Group 1 (93.99 ± 13.91 kg vs. 75.29 ± 10.70 kg; *p* < 0.001). No significant differences were observed between the groups in terms of height (*p* = 0.078). The sociodemographic characteristics can be seen in Table 1.

Regarding the MCT variables related to balance and muscle reaction speed, no statistically significant differences were observed between groups in the composite MCT and Z MCT scores (*p* = 0.208 for both), nor in the forward direction (*p* = 0.298). Only the backward direction of the MCT showed a significant difference, with lower values in Group 2 (135.83 ± 7.75) compared to Group 1 (140.05 ± 10.44; *p* = 0.041). These results should be interpreted with caution, as non-significant findings do not imply the absence of group differences, but rather that any such differences could not be statistically confirmed in this sample.

Similarly, no significant group differences were found in muscle strength assessed by dynamometry for the upper or lower limbs. Specifically, average and maximum strength values of the right and left arms (*p* > 0.18), back muscles (*p* = 0.06), and lower limbs (*p* > 0.41) did not reach statistical significance. These results suggest comparable performance between groups in isolated strength measures, and further research with larger sample sizes may be needed to clarify potential trends.

In contrast, the Dynamic Strength Index (DSI) was significantly lower in Group 2 (3.77 ± 0.57) than in Group 1 (4.58 ± 0.55; *p* < 0.001). Similarly, both energy expenditure (mean METS) and physical activity level (PAL) were significantly reduced in Group 2 (*p* < 0.001), supporting the relationship between higher fat mass and lower overall physical performance.

The results of the variable measures can be seen in Table 2.

## 4. Discussion

This study sought to investigate the relationship between the PAL and body composition and the levels of psychomotor performance and strength in men. The main findings of this investigation were that Group 1 was more physically active (in terms of energy expenditure) than Group 2, with a statistically significant difference (*p* < 0.001) and averages of 1.65 and 1.4, respectively. This may be related to the groups’ fat percentages and average METS.

With regard to strength data from dynamometry, there was no significance between the groups in the average and maximum strength of the right arm (*p* = 0.184), left arm (*p* = 0.308), and lower limbs (*p* = 0.419). However, regarding the DSI, there was a significant difference between Group 1 and Group 2, with Group 2 being significantly lower (*p* < 0.001). Regarding motor control, there was no significant difference between the groups in compound MCT (*p* = 0.208) and the average total forward MCT (*p* = 0.298). However, there was a significant difference in the average total backward MCT (*p* = 0.041), where Group 1 had higher values.

Women were excluded to reduce variability related to hormonal fluctuations and sex-based differences in body composition and neuromuscular function. While this improved sample homogeneity, it limits the generalizability of the results. Future studies should include female participants to explore potential sex-specific associations.

Research on this subject is becoming increasingly present and necessary in current times, marked by worldwide trends, in all regions of the globe, of increased sedentary behavior, lower PAL, and growing numbers of overweight and obesity accompanied by sarcopenia (loss of muscle mass) with age [11]. Based on the fat percentage cut-off point established for this sample, Group 1 was made up of individuals with percentages considered good, healthy, while Group 2 had poor percentages, indicating excess fat.

At this stage of life, PAL plays a fundamental role in controlling and reducing the percentage of fat, and in gaining and maintaining muscle mass, which are important indicators of health and risk for various contemporary chronic diseases [23]. Onofrei & Amaricai [24] agree with our findings, postulating that higher PAL has a positive impact on adequate body composition.

Body composition, together with fat percentage and PAL, significantly affects strength, motor coordination, and postural control [25]. Excess body fat contributes to a reduction in balance and motor control, and physical inactivity aggravates these effects by promoting a reduction in muscle mass and a deterioration in human performance in psychomotor aspects [26]. De Oliveira Neto et al. [27] point out that eutrophic individuals have better body functionality, better metabolic indicators, and lower chances of sarcopenia (progressive loss of muscle mass) with advancing age.

Regarding the values obtained in Dynamometry, they reflect the speed with which the neuromuscular system perceives the postural disturbance generated by the equipment and activates the muscles needed to stabilize the body, indicating the individual’s neuromuscular response score to movement [28]. The MCT is a test carried out on a moving platform, in which the individual’s balance is disturbed in all directions and the capacity of the neuromuscular system to react and respond in a motor way is assessed in terms of range of movement and reaction speed of muscle contraction [20].

It was expected that the higher the fat percentage, when the equipment platform moves backwards, it would generate a forward body imbalance, and that obese individuals, due to the accumulation of anteriorized fat, predominantly in the abdomen, would naturally have a more forward postural projection. This may be the reason why individuals with a higher fat mass take less time to react, because when they move forward, they move away from the base of support more quickly than eutrophic individuals.

This leads to the conclusion that, based on the results, the higher their percentage of fat, the less active and less DSI they tend to be. Despite the lack of significant differences in average and maximum strength levels across the legs, arms, and trunk among the groups, our findings suggest that muscle strength may not be exclusively associated with PAL or body composition alone. Physically active individuals do not necessarily exhibit greater strength or balance, as activity can stimulate different physical capacities without directly influencing maximal strength [29].

Additionally, individuals with a higher fat percentage tend to have a lower center of gravity, which may reduce their margin of imbalance and influence postural stability [30]. These factors highlight the complexity of the relationship between strength, PAL, and body composition, emphasizing the need for multifactorial approaches to understanding physical performance and functionality.

Maintaining a lower fat mass and an adequate level of strength are essential factors for daily functionality, contributing to the efficient performance of activities such as walking, climbing stairs, and carrying objects [31]. Moreover, adequate strength levels and higher PAL are directly associated with improved quality of life, enhancing autonomy and reducing the risk of metabolic and musculoskeletal disorders [32]. This highlights the importance of strategies that promote both fat reduction and muscle strength development to optimize health and functionality throughout life.

The results highlight reflections and implications for multidisciplinary professional practice around health regarding the need for public and private health interventions and programs aimed at increasing physical activity and promoting lifestyle changes in the general population and, specifically, in overweight and obese individuals. This is crucial for preventing health problems related to excess weight, sarcopenia (loss of muscle mass), and falls (due to impaired balance), as well as increasing functional capacity during the aging process. Providing greater levels of strength, balance, and better psychomotor performance, fundamental psychophysiological capacities when thinking about longevity with quality of life and well-being.

This study has some limitations: its cross-sectional nature does not allow causal relationships to be inferred, only associations. The limited number of participants and the fact that it is a sample of adults exclusively from one region of Spain may restrict the representativeness and generalizability of the results. In addition, not considering variations such as occupation and daily activities can introduce biases, since occasional physical exercise at work or during domestic activities can have an impact on functional capacity. Occupational and lifestyle activities were not considered in this study, which may influence overall physical activity and functional capacity. Future research should account for these factors to provide a more comprehensive analysis. Future studies should consider longitudinal designs and interventions aimed at proving the associations found in this study. Additionally, considering larger and more diverse samples of this population will allow for a more comprehensive and robust analysis of the impact of physical activity and body composition on strength and psychomotor performance in adults. This, in turn, will guide multi-professional practices and enable more assertive health actions for this population.

## 5. Conclusions

In conclusion, our findings indicate that individuals with a higher percentage of body fat tend to have lower levels of strength, physical activity, and energy expenditure. They also have impaired balance and muscle contraction speed. The lower Strength Index in individuals with higher body fat highlights the potential negative impact of excess adiposity on overall physical performance. These results emphasize the need for more longitudinal studies with targeted practical interventions to deepen our understanding of the relationship between body composition and levels of strength and physical activity, which can have a positive impact on general health and quality of life.

## Figures and Tables

**Table 1:** Sociodemographic results.

	Group 1 (n = 32)	Group 2 (n = 32)	*p*-Value	Cohen’s d
Age	55.09 ± 5.40	55.28 ± 6.11	0.449	−0.033
Weight	75.29 ± 10.70	93.99 ± 13.91	<0.001	−1.507
Height	1.74 ± 0.05	1.76 ± 0.06	0.078	−0.362

Values are expressed as means ± standard deviation. *p*-values indicate the statistical significance of differences between groups.

**Table 2:** Results of muscle strength and balance/reaction speed.

	Group 1 (n = 32)	Group 2 (n = 32)	*p*-Value	Cohen’s d
Compound MCT	137.91 ± 7.29	136.31 ± 8.27	0.208	0.205
Z MCT	−0.10 ± 0.93	0.102 ± 1.06	0.208	−0.203
Average total return MCT	140.05 ± 10.44	135.83 ± 7.75	0.041	0.459
Average total forward MCT	140.5 ± 11.66	142.44 ± 14.38	0.298	−0.144
Average right arm strength	42.01 ± 6.71	42.8 ± 6.97	0.325	−0.115
Maximum right arm strength	43.72 ± 6.77	45.34 ± 7.52	0.184	−0.226
Average left arm strength	41.97 ± 6.41	42.16 ± 6.69	0.455	−0.029
Average back strength	98.96 ± 17.88	105.93 ± 17.51	0.06	−0.394
Maximum back strength	105.56 ± 17.30	112.46 ± 17.75	0.06	−0.394
Average lower limb	139.04 ± 24.79	140.03 ± 27.73	0.44	−0.038
Maximum lower limb strength	149.87 ± 25.36	148.48 ± 28.79	0.419	0.051
Dynamic Strength Index (DSI)	4.58 ± 0.55	3.77 ± 0.57	<0.001	1.446
Average METS	1.65 ± 0.18	1.4 ± 0.16	<0.001	1.468
AMIGO	1.65 ± 0.18	1.39 ± 0.15	<0.001	1.569

Values are expressed as means ± standard deviation. MCT = Measurement of Total Composition; METS = Metabolic Equivalents of Task; AMIGO = Physical Activity Index. *p*-values indicate the statistical significance of differences between groups.

## Data Availability

The data presented in this study are available upon request from the first author. Data are unavailable due to privacy or ethical restrictions.

## Abbreviations

DXA	Dual-Energy X-ray Absorptiometry
MCT	Motor Control Test
DSI	Dynamic Strength Index
PAL	Physical Activity Level
BMI	Body Mass Index
MET	Metabolic Equivalent of Task
PRONAF	Nutritional and Physical Activity Program for Obesity Control
SWA	SenseWear^®^ Armband
SPSS	Statistical Program for Social Science
