# Correction: Delfa-de-la-Morena et al. Relationship of Physical Activity Levels and Body Composition with Psychomotor Performance and Strength in Men. *Healthcare* 2025, *13*, 1789

**DOI:** 10.3390/healthcare13232996

**Published:** 2025-11-21

**Authors:** José Manuel Delfa-de-la-Morena, Pedro Pinheiro Paes, Frederico Camarotti Júnior, Rubem Cordeiro Feitosa, Débora Priscila Lima de Oliveira, Juan-José Mijarra-Murillo, Miriam García-González, Víctor Riquelme-Aguado

**Affiliations:** 1 Department of Physical Therapy, Occupational Therapy, Rehabilitation and Physical Medicine, Health Sciences Faculty, Rey Juan Carlos University, 28922 Madrid, Spain; jose.delfa@urjc.es (J.M.D.-d.-l.-M.); miriam.garciag@urjc.es (M.G.-G.); 2 Department of Physical Education, Federal University of Pernambuco, Recife 50670-901, Brazil; pppaes@ufpe.br (P.P.P.); frederico.camarottijunior@ufpe.br (F.C.J.); rubem.cordeiro@ufpe.br (R.C.F.); debora.limaoliveira@ufpe.br (D.P.L.d.O.); 3 Department of Basic Health Sciences, Health Sciences Faculty, Rey Juan Carlos University, 28922 Madrid, Spain; victor.riquelme@urjc.es

In the original publication [1], a coordination error occurred among the authors during submission, resulting in the incorrect assignment of the corresponding author. The correct corresponding author is Juan-José Mijarra-Murillo, instead of Miriam García-González. The authors state that the scientific conclusions are unaffected. This correction was approved by the Academic Editor. The original publication has also been updated.
